# Author Correction: In vitro reconstitution of *Escherichia coli* divisome activation

**DOI:** 10.1038/s41467-022-34485-1

**Published:** 2022-11-08

**Authors:** Philipp Radler, Natalia Baranova, Paulo Caldas, Christoph Sommer, Mar López-Pelegrín, David Michalik, Martin Loose

**Affiliations:** 1 Institute for Science and Technology Austria (IST Austria), Klosterneuburg, Austria; 2 University of Vienna, Department of Pharmaceutical Sciences, Vienna, Austria; 3 UCIBIO—Applied Molecular Biosciences Unit, Department of Life Sciences, NOVA School of Science and Technology, Universidade Nova de Lisboa, Caparica, Portugal

**Keywords:** Cytoskeletal proteins, Bacteria, Biochemical networks, Confocal microscopy

Correction to: *Nature Communications* 10.1038/s41467-022-30301-y, published online 12 May 2022

The original version of this Article contained an error in Fig. 3b, in which wrong single molecule residence times have been reported. This has been corrected in both the PDF and HTML versions of the Article.

To further amend this error, the following corrections in both the PDF and HTML versions of the Article have been made:

1. The original caption of Fig. 3b incorrectly read “b. The mobility of both proteins decreases with increasing concentrations, but R286W remains 10x more mobile and has a 2-10x lower lifetime”. The correct version replaces this sentence with “b. With higher concentrations the mobility of both proteins decreases and the residence time increases, but R286W remains 10x more mobile and has a 2-7x lower lifetime”.
2. The second paragraph of the section entitled “FtsNcyto does not depolymerize FtsA WT oligomers and enhances interaction of FtsA R286W” within the Results incorrectly read ‘At 0.1 μM, we found that FtsA WT showed a low mobility with a diffusion coefficient of 0.14 ± 0.04 μm^2^/s and a mean residence time on the membrane of 10.2 ± 0.7 s. With increasing bulk concentrations and protein densities, the membrane residence time remained constant (9.4 ± 0.3 s at 0.8 μM FtsA), while the diffusion coefficient dropped to a value of 0.004 ± 0.001 μm^2^/s indicating almost immobile proteins on the membrane. Similar to previous FRAP experiments in vivo^18^, we found a faster exchange of FtsA R286W with a single molecule residence time 2–10 fold shorter than that of wild-type FtsA (from 0.5 ± 0.1 s to 4.8 ± 0.5 s for 0.1 and 0.8 μM respectively).’. The correct version states ‘At 0.1 μM, we found that FtsA WT showed a low mobility with a diffusion coefficient of 0.14 ± 0.04 μm^2^/s and a mean residence time on the membrane of 19.4 ± 3.9 s. With increasing bulk concentrations and protein densities, the membrane residence time increased (35.1 ± 2.0 s at 0.8 μM FtsA), while the diffusion coefficient dropped to a value of 0.004 ± 0.001 μm^2^/s indicating almost immobile proteins on the membrane. Similar to previous FRAP experiments in vivo^18^, we found a faster exchange of FtsA R286W with a single molecule residence time 2–7 fold shorter than that of wild-type FtsA (from 2.6 ± 0.5 s to 14.1 ± 2.3 s for 0.1 and 0.8 μM respectively)’.
3. The link https://github.com/paulocaldas/PhotobleachingCorrectionSPT, which contains the updated and corrected code to quantify single molecule lifetime, has been added to the Code availability statement.

Furthermore, the original version of the Supplementary Information associated with this Article contained errors in Supplementary Figure 1e and in the Source Data file, in which wrong single molecule residence times have been provided as well. The HTML has been updated to include corrected versions of the Supplementary Information and the Source Data file.

## Supplementary information

Updated Supplementary Information

## Source data

Updated Source Data File

