# Supplementary material for: In vitro reconstitution of Escherichia coli divisome activation
Source: Nat Commun. 2022 May 12;13:2635. doi: 10.1038/s41467-022-30301-y (PMC9098913; doi:10.1038/s41467-022-30301-y)
Supplement: Supplementary file 1 — Supplementary Information [file 41467_2022_30301_MOESM1_ESM.pdf]

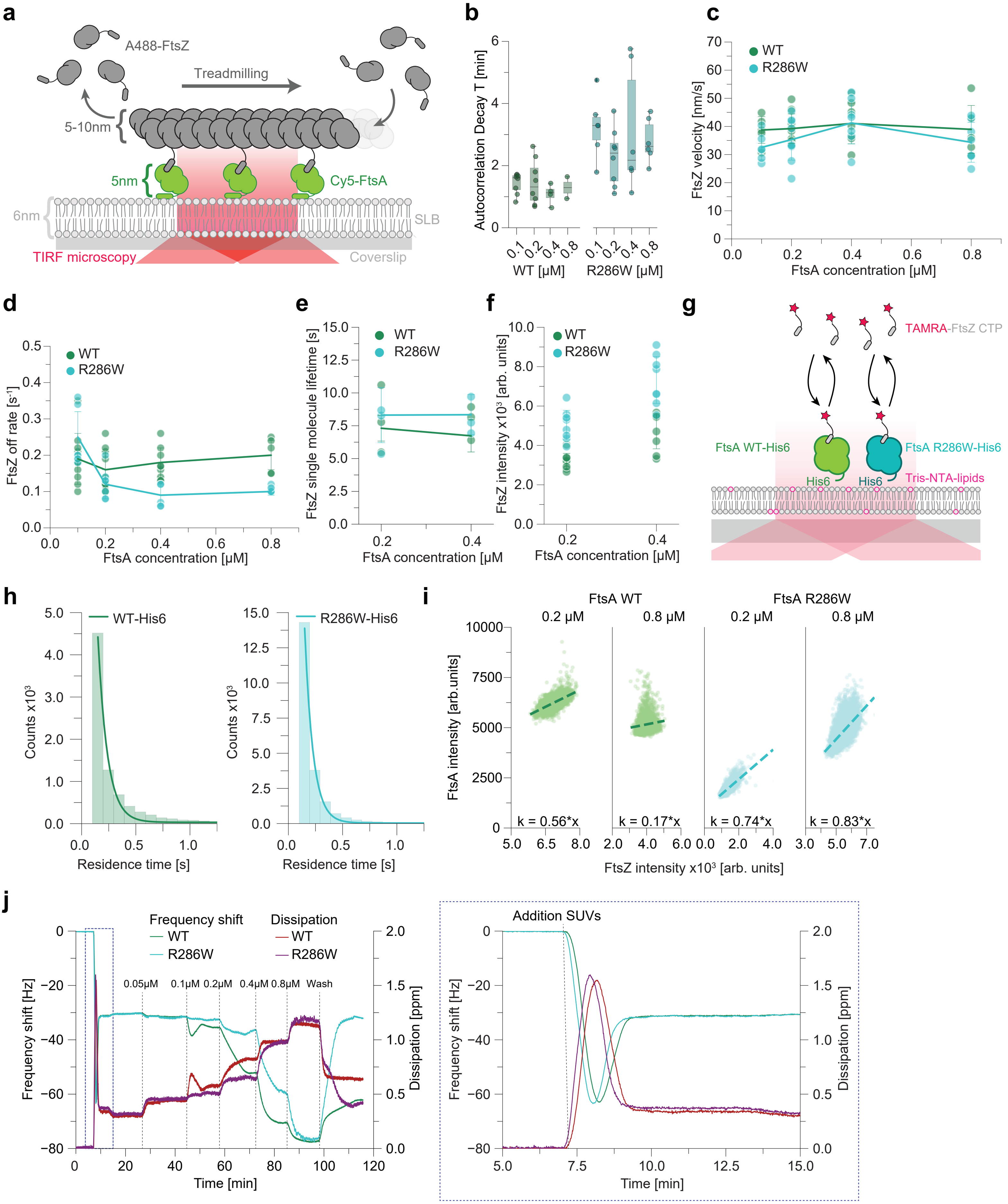
a
A488-FtsZ
Treadmilling
5-10nm
5nm
Cy5-FtsA
6nm
SLB
TIRF microscopy
Coverslip
b
Autocorrelation Decay T [min]
WT [μM]
R286W [μM]
0.1 0.2 0.4 0.8
c
WT
R286W
FtsZ velocity [nm/s]
FtsA concentration [μM]
d
FtsZ off rate [s-1]
FtsA concentration [μM]
e
FtsZ single molecule lifetime [s]
FtsA concentration [μM
f
FtsZ intensity x10³ [arb. units]
FtsA concentration [μM]
g
TAMRA-FtsZ CTP
FtsA WT-His6
FtsA R286W-His6
His6
His6
Tris-NTA-lipids
h
WT-His6
R286W-His6
Counts x10³
Residence time [s]
i
FtsA WT
FtsA R286W
0.2 μM
0.8 μM
0.2 μM
0.8 μM
FtsA intensity [arb.units]
k = 0.56*x
k = 0.17*x
k = 0.74*x
k = 0.83*x
FtsZ intensity x10³ [arb. units]
j
Frequency shift
WT
R286W
Dissipation
WT
R286W
0.05μM
0.1μM
0.2μM
0.4μM
0.8μM
Wash
Frequency shift [Hz]
Dissipation [ppm]
Time [min]
Addition SUVs
Frequency shift [Hz]
Dissipation [ppm]
Time [min]

Figure S1: FtsZ dynamics are identical with FtsA WT and R286W, but more FtsZ is recruited by FtsA R286W

a, Schematic depicting the experiment setup and the corresponding dimensions of the proteins. **b,** The persistency of the FtsZ pattern is lower with FtsA WT compared to R286W at all tested concentrations, indicated by higher autocorrelation decay τ. n(WT) = 8/8/4/2 and n(R286W) = 6/8/6/6 for increasing FtsA concentrations. **c,** FtsZ treadmilling velocity is identical with FtsA WT or R286W. **d,** At lower FtsA concentrations, the off-rate of FtsZ is similar to FtsA WT and R286W. At 0.8 µM, FtsZ remains bound longer with R286W. The number of independent biological replicates for **c, d** are n=5/9/7/5 for increasing FtsA concentrations. **e,** Single molecule lifetimes of FtsZ are similar at 0.2 µM and 0.4 µM FtsA (n=3). **f,** Intensity of membrane bound FtsZ is higher in the presence of FtsA R286W (n=8 for each concentration and protein). **g,** Scheme of TIRF experiment to measure the lifetime of the TAMRA labelled FtsZ-C-terminal-peptide. **h,** Representative histograms of the FtsZ CTP lifetime distribution with FtsA-His6 and R286W-His6. **i,** Scatter plots of FtsA WT (green) and R286W (cyan) intensities against FtsZ intensities from colocalization analysis at 0.2 and 0.8 µM. The steepness of the slope is consistently high for FtsA R286W but decreases drastically for FtsA WT at 0.8 µM. The plots are representative experiments, performed on the same day to ensure comparable intensity values. **j,** Representative curves from QCM-D experiments showing changes in resonance frequency during the sequential addition of increasing concentrations of FtsA. At 0.8 µM the frequency change (=membrane binding) is identical between FtsA WT (green) and R286W (cyan). R286W can be washed easily from the membrane, whereas WT remains bound to the membrane. The zoom-in on the right side shows the initial stages of the QCM-D experiment and the formation of a SLB. Dots in all plots (except **S1i**) represent independent experiments, thick lines indicate the mean and error bars depict the standard deviation. The boxes indicate the 25–75th percentiles, whiskers show the maximum/minimum values within the standard deviation, the midline indicates the median value and diamonds indicate outliers. Source data are provided as a Source Data file.

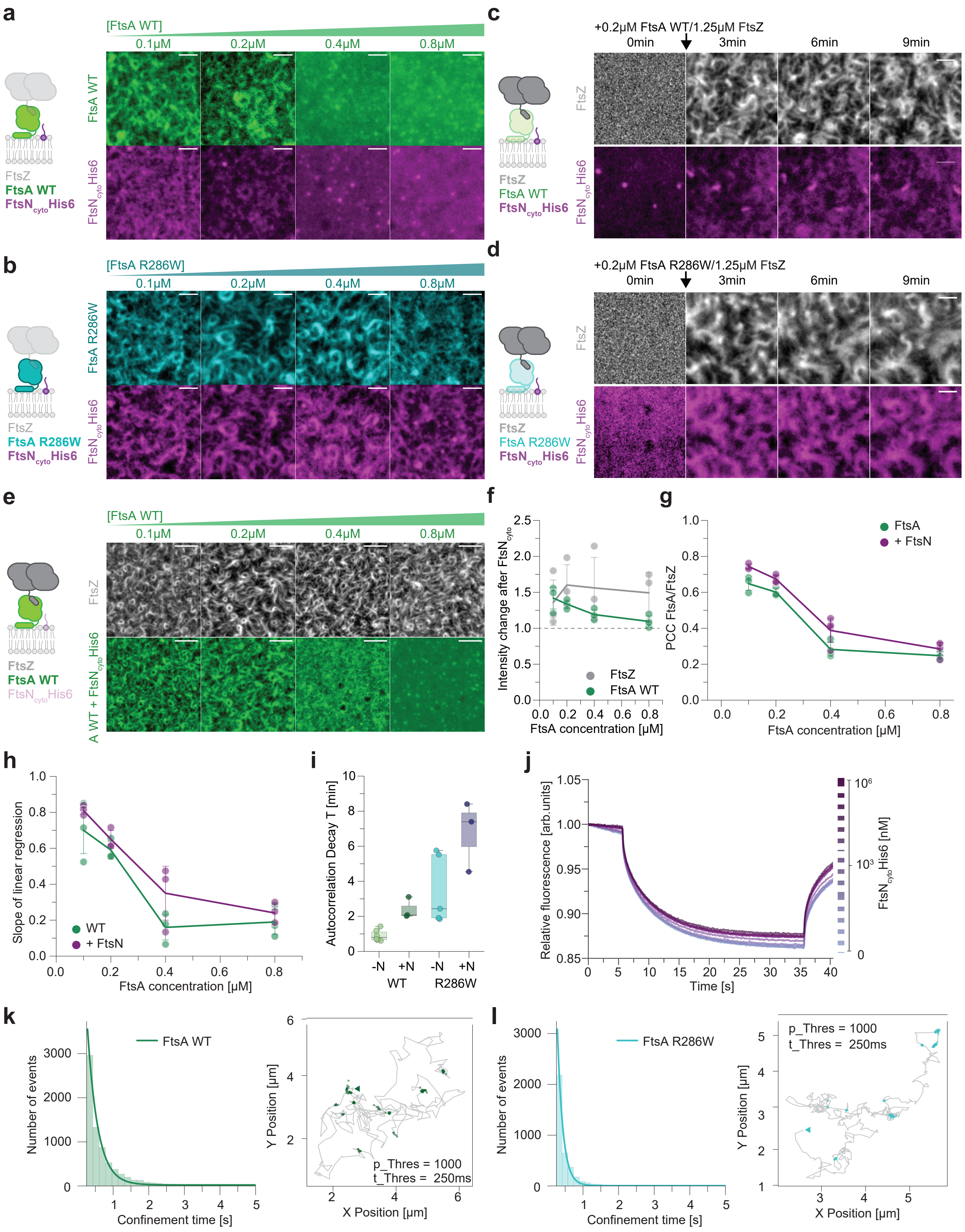

a
[FtsA WT]
0.1μM
0.2μM
0.4μM
0.8μM
FtsA WT
FtsN_cyto His6
FtsZ
FtsA WT
FtsN_cyto His6
b
[FtsA R286W]
0.1μM
0.2μM
0.4μM
0.8μM
FtsA R286W
FtsN_cyto His6
FtsZ
FtsA R286W
FtsN_cyto His6
c
+0.2μM FtsA WT/1.25μM FtsZ
0min
3min
6min
9min
FtsZ
FtsN_cyto His6
FtsZ
FtsA WT
FtsN_cyto His6
d
+0.2μM FtsA R286W/1.25μM FtsZ
0min
3min
6min
9min
FtsZ
FtsN_cyto His6
FtsZ
FtsA R286W
FtsN_cyto His6
e
[FtsA WT]
0.1μM
0.2μM
0.4μM
0.8μM
FtsZ
A WT + FtsN_cyto His6
FtsZ
FtsA WT
FtsN_cyto His6
f
Intensity change after FtsN_cyto
FtsA concentration [μM]
FtsZ
FtsA WT
g
PCC FtsA/FtsZ
FtsA concentration [μM]
FtsA
+ FtsN
h
Slope of linear regression
FtsA concentration [μM]
WT
+ FtsN
i
Autocorrelation Decay T [min]
-N
+N
-N
+N
WT
R286W
j
Relative fluorescence [arb.units]
Time [s]
FtsN_cyto His6 [nM]
k
Number of events
Confinement time [s]
FtsA WT
Y Position [μm]
X Position [μm]
p_Thres = 1000
t_Thres = 250ms
l
Number of events
Confinement time [s]
FtsA R286W
p_Thres = 1000
t_Thres = 250ms
Y Position [μm]
X Position [μm]

Figure S2: $FtsN_{cyto}$ follows FtsA R286W co-filaments better than FtsA WT

a/b, Representative micrographs of Cy3-FtsA WT (green) and Cy5-$FtsN_{cyto}$ (magenta) **(a)** or Cy3-FtsA R286W (cyan) and Cy5-$FtsN_{cyto}$ (magenta) **(b)** at increasing FtsA concentrations with FtsZ and 0.25% Tris-NTA-lipids. **c/d,** Starting from a homogeneous distribution, Cy5-$FtsN_{cyto}$ (magenta) colocalizes with FtsZ filaments on the membrane after the addition of Alexa488-FtsZ (grey) and 0.2 µM FtsA WT **(c)** or 0.2 µM FtsA R286W **(d)** at 0 min. Schematics on the left side of **a-d** indicate all components present in the assay and bold labels indicate fluorescent components. Scale bars are 2 µm. **e,** Representative micrographs of Cy5-FtsA WT (green) and Alexa488-FtsZ (grey) with $FtsN_{cyto}$. Scale bars are 5 µm. **f,** $FtsN_{cyto}$ increases the amount of recruited FtsZ, whereas the amount of membrane-bound FtsA changes only slightly above 0.2 µM. **g,** Colocalization of FtsA/FtsZ quantified by PCC shows that colocalization is slightly increased with $FtsN_{cyto}$. **h,** The slope of the linear regression indicates the [FtsZ] vs. [FtsA] ratio. With $FtsN_{cyto}$, the slope for FtsA WT increases slightly. The measurements in **f-h** were replicated three times. **i,** $FtsN_{cyto}$ increases the persistency of the FtsZ pattern indicated by an increase in the autocorrelation decay time τ. n(WT) = 6, n(WT+FtsN) =3, n(R286W)=5, n(R286W+FtsN)=3 **j,** Representative MST traces for a titration of $FtsN_{cyto}$ to 50nM Cy5-FtsA WT. MST measurements were repeated three times for WT and R286W. **k, l,** Histogram of $FtsN_{cyto}$ confinement times to co-filaments of FtsZ and FtsA WT (**k**) or FtsA R286W (**l**). Representative tracks containing $FtsN_{cyto}$ confinement events in the presence of FtsA WT (green) and R286W (cyan) are shown next to the histograms. Arrowheads indicate the beginning of the tracks. Insets indicate the filters for the analysis. Dots in all plots represent independent experiments, thick lines indicate the mean and error bars depict the standard deviation. The boxes indicate the 25–75th percentiles, whiskers show the maximum/minimum values within the standard deviation and the midline indicates the median value. Source data are provided as a Source Data file.

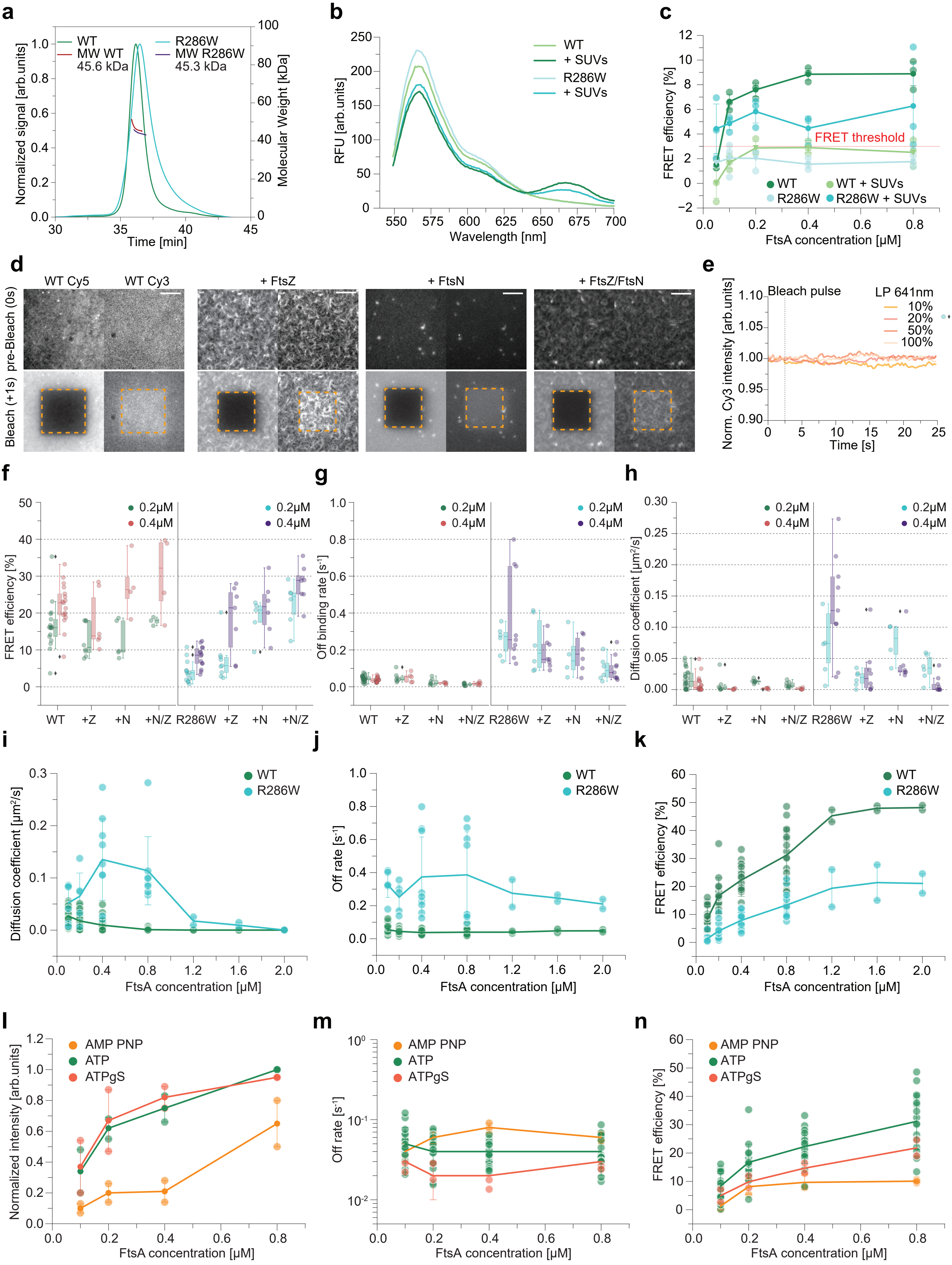

a
WT
MW WT 45.6 kDa
R286W
MW R286W 45.3 kDa
Normalized signal [arb.units]
Molecular Weight [kDa]
Time [min]
b
WT
+ SUVs
R286W
+ SUVs
RFU [arb.units]
Wavelength [nm]
c
FRET efficiency [%]
FRET threshold
WT
WT + SUVs
R286W
R286W + SUVs
FtsA concentration [μM]
d
WT Cy5
WT Cy3
+ FtsZ
+ FtsN
+ FtsZ/FtsN
pre-Bleach (0s)
Bleach (+1s)
e
Bleach pulse
LP 641nm
10%
20%
50%
100%
Norm. Cy3 intensity [arb.units]
Time [s]
f
FRET efficiency [%]
0.2μM
0.4μM
WT
+Z
+N
+N/Z
R286W
g
Off binding rate [s-1]
h
Diffusion coefficient [μm²/s]
i
Diffusion coefficient [μm²/s]
WT
R286W
FtsA concentration [μM]
j
Off rate [s-1]
k
FRET efficiency [%]
l
AMP PNP
ATP
ATPgS
Normalized intensity [arb.units]
m
Off rate [s-1]
n
FRET efficiency [%]

Figure S3: FtsA WT self-interaction is membrane dependent, but does not require ATP hydrolysis

a, SEC-MALLS experiments show that FtsA WT and R286W are monomers in solution. **b,** Representative spectra of cuvette-FRET measurements indicate that self-interaction of FtsA WT (green) and R286W (cyan) depends on the presence of membranes. **c,** With lipids FtsA WT exhibits stronger FRET, which is due to the lower membrane residence time and the previously proposed less ordered arrangements of FtsA R286W. n=3 for each condition. **d,** Representative micrographs for acceptor photobleaching experiments of FtsA WT alone, + FtsZ, + $FtsN_{cyto}$ and + FtsZ/$FtsN_{cyto}$. Scale bars are 5 μm. **e,** The intensity of membrane-bound Cy3-FtsA WT is not affected by a Cy5-bleach pulse with increasing laser power (LP). **f-h**, Boxplots showing FRET efficiency **(f)**, off-binding rate **(g)** and the diffusion coefficient **(h)** for better comparison of FtsA WT and FtsA R286W at 0.2 or 0.4 μM alone. **i,** Lateral diffusion of FtsA R286W drops significantly above 0.8 μM. The lateral mobility of R286W seems lower at low concentrations, as the fast off-binding rate dominates. **j,** The off-rate of FtsA R286W is faster than that of WT. **k,** FRET efficiency of FtsA R286W and FtsA WT saturates at concentrations above 0.8 μM. n(WT) = 19/17/17/17/2/2/2 and n(R286W) = 11/13/14/13/2/2/2 for increasing FtsA concentrations. **l,** Nucleotide hydrolysis is not important for membrane binding of FtsA WT, as the protein binds to SLBs comparable in the presence of ATP and ATPγS. Membrane binding is decreased in the presence of AMP PNP. **m,** The off-rate of FtsA WT is similar in the presence of ATP or ATPγS. **n,** FRET efficiency of FtsA WT is similar in the presence of ATP or ATPγS. Dots in represent independent experiments, thick lines indicate the mean and error bars depict the standard deviation. The boxes indicate the 25–75th percentiles, whiskers show the maximum/minimum values within the standard deviation, the midline indicates the median value and diamonds indicate outliers. Source data are provided as a Source Data file.

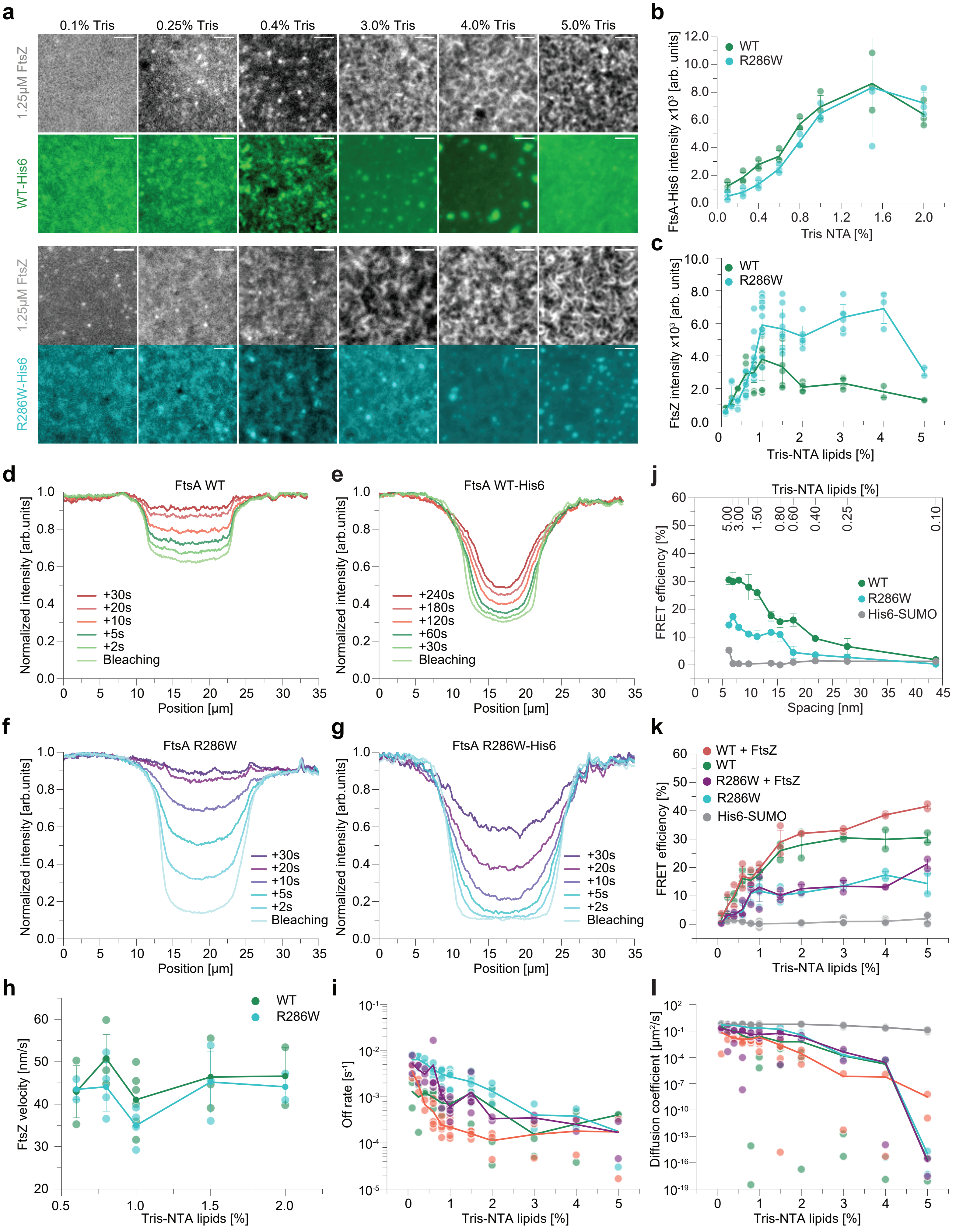

a
0.1% Tris
0.25% Tris
0.4% Tris
3.0% Tris
4.0% Tris
5.0% Tris
1.25µM FtsZ
WT-His6
1.25µM FtsZ
R286W-His6
b
WT
R286W
FtsA-His6 intensity x10³ [arb. units]
Tris NTA [%]
c
WT
R286W
FtsZ intensity x10³ [arb. units]
Tris-NTA lipids [%]
d
FtsA WT
+30s
+20s
+10s
+5s
+2s
Bleaching
Normalized intensity [arb.units]
Position [µm]
e
FtsA WT-His6
+240s
+180s
+120s
+60s
+30s
Bleaching
Normalized intensity [arb.units]
Position [µm]
j
Tris-NTA lipids [%]
5.00
3.00
1.50
0.80
0.60
0.40
0.25
0.10
WT
R286W
His6-SUMO
FRET efficiency [%]
Spacing [nm]
f
FtsA R286W
+30s
+20s
+10s
+5s
+2s
Bleaching
Normalized intensity [arb.units]
Position [µm]
g
FtsA R286W-His6
+30s
+20s
+10s
+5s
+2s
Bleaching
Normalized intensity [arb.units]
Position [µm]
k
WT + FtsZ
WT
R286W + FtsZ
R286W
His6-SUMO
FRET efficiency [%]
Tris-NTA lipids [%]
h
WT
R286W
FtsZ velocity [nm/s]
Tris-NTA lipids [%]
i
Off rate [s⁻¹]
Tris-NTA lipids [%]
l
Diffusion coefficient [µm²/s]
Tris-NTA lipids [%]

Figure S4: FtsZ pattern forms more efficient with FtsA R286W-His6, even at high densities
a, Representative micrographs of Alexa488-FtsZ (grey) and Cy5-FtsA-His6 (green) or Cy5-FtsA R286W-His6 (cyan) at Tris-NTA lipid densities not shown in **Fig 4**. FtsZ filaments form a more disrupted cytoskeletal pattern at high densities of FtsA-His6. Scale bars are 2 µm. **b,** Using His-tagged-proteins and Tris-NTA-lipids allows to control the density of FtsA as demonstrated by increasing intensities of His-tagged FtsAs until 1.5% Tris-NTA, where saturation is reached. n =3 for each Tris-NTA lipid density. **c,** At Tris-NTA lipid densities higher than 1%, FtsA R286W-His6 is able to recruit more FtsZ filaments than FtsA-His6. n(WT-His6) = 2/3/3/10/11/12/7/7/4/2/2 and n(R286W-His6) = 6/6/5/12/13/12/9/9/6/3/3 for increasing Tris-NTA densities. **d-g,** Comparison of FRAP recovery profiles of native FtsAs **(d, f)** and His-tagged FtsAs **(e, g)**. The recovery of the native proteins is dominated by exchange, while His-tagged proteins recover dominantly by lateral diffusion, as can be seen by the different shapes of the recovery profiles. **h,** The treadmilling speed of FtsZ is similar at different densities of FtsA-His6 or R286W-His6. I **i,** As expected, off-rates of FtsA-His6 and FtsA R286W-His6 obtained from FRAP experiments are close to zero. **j**, The measured FRET of His-tagged FtsAs and the theoretical spacing is shown. Importantly FRET is already measured before the theoretical FRET border is reached (5nm), indicating active self-interaction. The His6-SUMO control only exhibits weak FRET at the maximum density. **k,** FRET efficiency of His-tagged FtsAs ± FtsZ and His6-SUMO on membranes with up to 5% Tris-NTA lipids. **l,** Diffusion coefficient of His-tagged FtsAs ± FtsZ and His6-SUMO on membranes with up to 5% Tris-NTA lipids (n=2/2/2/4/5/4/5/4/3/2/2 for increasing Tris-NTA densities). Dots represent independent experiments, thick lines indicate the mean and error bars depict the standard deviation. Source data are provided as a Source Data file.

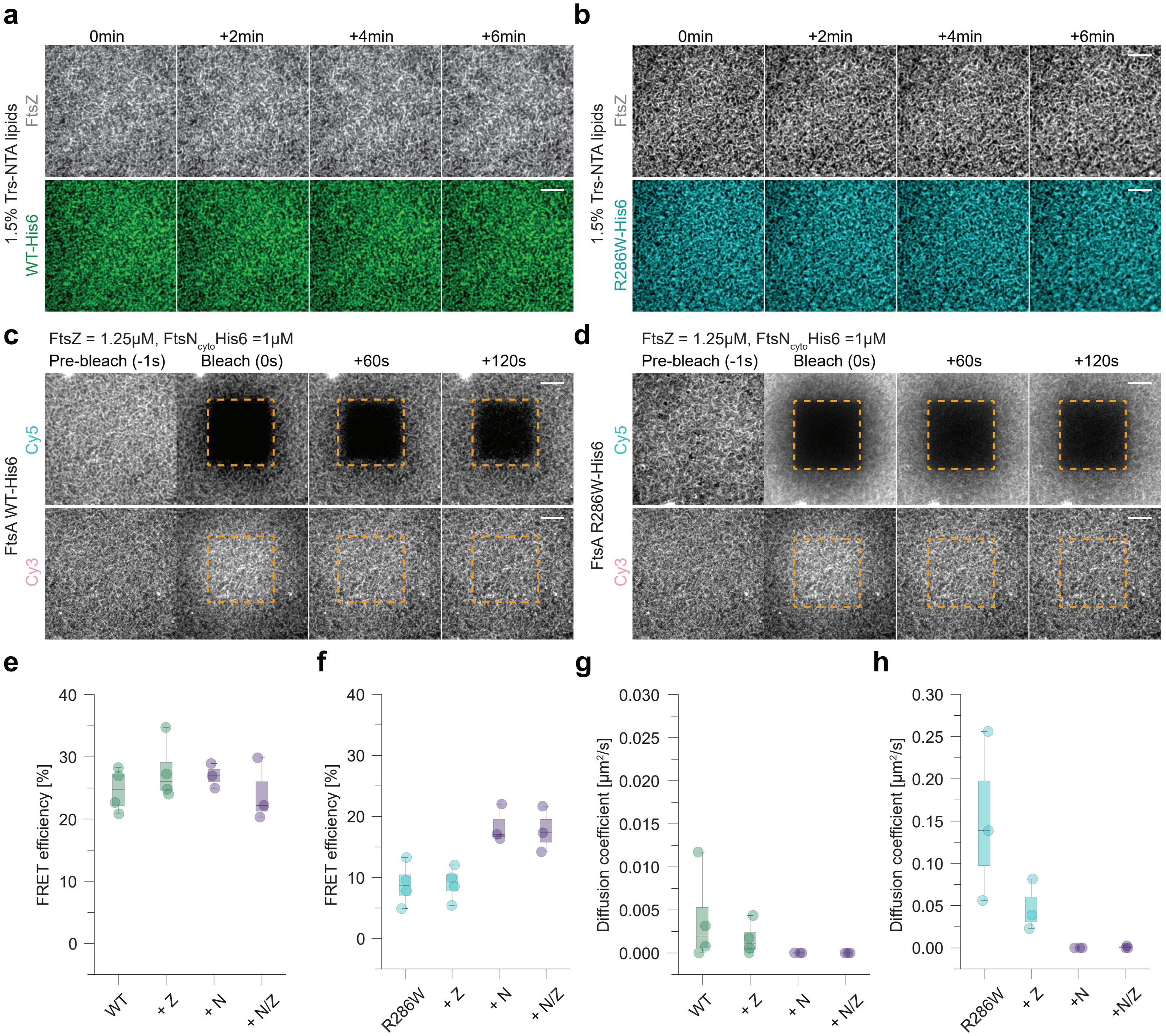
a
0min
+2min
+4min
+6min
1.5% Trs-NTA lipids
FtsZ
WT-His6
b
R286W-His6
c
FtsZ = 1.25μM, FtsN$_{cyto}$His6 =1μM
Pre-bleach (-1s)
Bleach (0s)
+60s
+120s
FtsA WT-His6
Cy5
Cy3
d
FtsA R286W-His6
e
FRET efficiency [%]
WT
+ Z
+ N
+ N/Z
f
R286W
g
Diffusion coefficient [μm²/s]
h
+N
+N/Z

Figure S5: FtsN$_{cyto}$ increases FRET of FtsA R286W-His6, and renders FtsA-His6 immobile

a, b, Representative micrographs of Alexa488-FtsZ (grey) and Cy5-FtsA-His6 (green**, a**) or Cy5-FtsA R286W-His6 (cyan**, b**) in the presence of FtsN$_{cyto}$ on bilayers with 1.5% Tris-NTA lipids. The FtsA/FtsZ co-filaments are static, as shown by snapshots taken every two minutes in a timelapse experiment. **c, d,** Representative micrographs of acceptor bleaching recovery and donor intensity increase of FtsA-His6 **(c)** and FtsA R286W-His6 **(d)** with non-labeled FtsZ and FtsN$_{cyto}$. The bleached area does not recover within 2 minutes. **e, f,** FRET of His-tagged FtsAs in the presence of FtsZ and FtsN$_{cyto}$. FRET signal of FtsA-His6 remains stable in all conditions tested **(e)**, while the measured FRET of FtsA R286W-His6 **(f)** increases twofold in the presence of FtsN$_{cyto}$. **g, h,** The diffusion of FtsA-His6 **(g)** and FtsA R286W-His6 **(h)** and decreases drastically in the presence of FtsN$_{cyto}$. Experiments with FtsAs alone and with FtsZ have been repeated four times. Experiments with FtsN$_{cyto}$ have been repeated three times. Scale bars are 5µm. All experiments were performed on SLBs containing 1.5% Tris NTA lipids. Precisely defining the density of membrane-bound proteins is difficult, as His-tagged FtsAs and FtsN$_{cyto}$ compete for binding to Tris-NTA lipids. Dots in all plots represent independent experiments. The boxes indicate the 25–75th percentiles, whiskers show the maximum/minimum values within the standard deviation and the midline indicates the median value. Source data are provided as a Source Data file.

Supplementary Table 1_Summary of Statistics of Figure 3 (FRET, Off-binding, Diffusion coefficient)

		FtsA WT			***FtsA R286W***			***Statistics***	***FtsA WT***			***FtsA R286W***			***Statistics***	***FtsA WT***			***FtsA R286W***			***Statistics***
		**FRET Efficiency [%]**							**Off binding rate [s^{-1}]**							**Diffusion coefficient [$\mu m^2/s$]**						
Concentration	Condition	mean	sd	n	mean	sd	n	p-Value	mean	sd	n	mean	sd	n	p-Value	mean	sd	n	mean	sd	n	p-Value
0.1µM	Alone	8.11	4.43	19	1.34	1.19	11	3.66E-06	0.05	0.03	18	0.33	0.08	6	1.73E-11	0.03	0.02	18	0.05	0.03	6	3.67E-02
	+FtsZ	7.85	3.79	10	2.26	0.50	2	8.64E-02	0.04	0.01	8	0.20	0.13	4	8.97E-03	0.02	0.01	8	0.01	0.01	4	1.26E-01
	+FtsN	8.56	2.85	5	9.24	6.60	2	8.84E-01	0.03	0.02	5	0.06	0.02	3	1.69E-01	0.02	0.01	5	0.07	0.10	3	3.01E-01
	+FtsN/Z	15.28	1.44	3	18.47	7.18	3	5.71E-01	0.02	0.01	5	0.09	0.03	3	1.92E-03	0.01	0.01	5	0.02	0.01	3	1.13E-01
0.2µM	Alone	16.45	6.33	17	4.11	2.88	13	7.95E-07	0.04	0.02	24	0.25	0.08	6	1.44E-12	0.02	0.02	24	0.15	0.20	6	3.98E-03
	+FtsZ	12.58	4.26	8	7.16	5.04	9	4.09E-02	0.05	0.03	7	0.23	0.14	6	1.03E-02	0.01	0.01	7	0.02	0.01	6	2.16E-01
	+FtsN	12.68	4.62	5	18.49	4.87	5	1.22E-01	0.03	0.02	7	0.17	0.11	4	1.48E-02	0.01	0.00	7	0.07	0.03	4	5.48E-04
	+FtsN/Z	17.79	0.96	3	22.39	5.52	6	2.47E-01	0.01	0.01	8	0.10	0.06	6	2.69E-03	0.01	0.00	8	0.04	0.02	6	3.82E-03
0.4µM	Alone	22.14	5.74	17	8.09	2.79	14	6.12E-09	0.04	0.01	18	0.37	0.24	9	6.73E-06	0.01	0.01	18	0.14	0.08	9	1.05E-06
	+FtsZ	17.74	7.31	6	18.19	8.78	7	9.28E-01	0.05	0.02	5	0.18	0.07	8	5.48E-03	0.00	0.00	5	0.03	0.04	8	1.56E-01
	+FtsN	27.29	7.13	4	21.27	6.98	6	2.70E-01	0.02	0.00	5	0.18	0.09	6	5.78E-03	0.00	0.00	5	0.05	0.04	6	3.55E-02
	+FtsN/Z	30.17	9.64	4	28.01	4.62	8	6.43E-01	0.02	0.01	6	0.10	0.07	9	1.22E-02	0.00	0.00	6	0.01	0.01	9	2.49E-01
0.8µM	Alone	30.63	9.86	17	13.36	4.59	13	1.81E-03	0.04	0.02	19	0.39	0.26	8	9.58E-03	0.00	0.00	19	0.11	0.07	8	2.57E-03
	+FtsZ	27.13	11.27	5	23.78	11.49	7	6.57E-01	0.06	0.02	3	0.16	0.07	8	4.84E-02	0.00	0.00	3	0.02	0.01	8	2.32E-02
	+FtsN	29.89	5.76	6	21.21	5.65	4	6.85E-02	0.03	0.01	4	0.19	0.13	3	9.35E-02	0.00	0.00	4	0.04	0.02	3	1.02E-01
	+FtsN/Z	34.22	1.13	3	30.62	5.58	6	3.62E-01	0.03	0.01	5	0.09	0.07	4	1.20E-01	0.00	0.00	5	0.01	0.00	4	1.14E-01

Supplementary Table 2_Reagents and Chemicals

Strain, Reagent or Resource	***Source***	***Identifier***
E. coli strains		
BL21 (DE3)		C2527
DH5α		18265017
Chemicals		
cOmplete™, EDTA-free Protease Inhibitor Cocktail	Roche Molecular Biochemicals	5056489001
DNase	ThermoFisher Scientific	90083
Potassium Chloride	Sigma-Aldrich	60130-1KG
Glycerol	Sigma-Aldrich	G5516
IPTG	Bartelt	6.259 683
Imidazole	Sigma-Aldrich	I2399
DTT,1,4-Dithiothreitol	Sigma-Aldrich	10708984001
(4-(2-hydroxyethyl)-1-piperazineethanesulfonic acid) (HEPES)	Sigma-Aldrich	H3375-25G
Piperazine-N,N-bis(2-ethanesulphonic acid) (PIPES)	Sigma-Aldrich	P6757
d-Desthiobiotin	Sigma-Aldrich	D1411
Strep-Tactin® Sepharose® resin	IBA-lifesciences	2-1201-002
HisPur Ni-NTA Resin; Thermo Fisher Scientific	ThermoFisher Scientific	88221
InstantBlue® Coomassie Protein Stain	abcam	ab119211
Hydrogen Peroxide H_2O_2	Sigma-Aldrich	216763
Sulfuric acid H_2SO_4 95-97%	Sigma-Aldrich	1007311000
β-mercaptoethanol	Sigma-Aldrich	M6250
HisPur^{TN} Ni-NTA resin	ThermoFisher Scientific	88221
7M Sortase	Addgene	Plasmid #51141
Tris(2-carboxyethyl)phosphine hydrochloride (TCEP)	Sigma-Aldrich	C4706
Protino Ni-TED resin	Lactan (Macherey Nagel)	74520030
Vivaspin 2 and 20 centrifugal concentrators 5-kDa	Sigma-Aldrich (Sartorius)	Z614181, Z614599
Catalase	Sigma Aldrich	C40
Glucose	Sigma Aldrich	G8270-1kg
GTP	ThermoFisher Scientific	R0461
ATP	ThermoFisher Scientific	R0441
Trolox (6-Hydroxy-2,5,7,8-tetramethylchromane-2-carboxylic acid)	Sigma Aldrich	238813
Glucose Oxidase	AL-Labortechnik (SERVA)	22778.01
Lipids		
DOPC (1,2 dioleoyl-sn-glycero-3-phosphocholine)	Avanti Polar Lipids	850375C
DOPG (1,2-dioleoyl-sn-glycero-3-phospho-(1'-rac-glycerol))	Avanti Polar Lipids	840475C
Tris-DODA-NTA	ApexMolecular	Custom

DOPE-Rhodamine (1,2-dioleoyl-*sn* -glycero-3-phosphoethanolamine-N-(lissamine rhodamine B sulfonyl)	Avanti Polar Lipids	810150C
Peptides		
$FtsN_{cyto}His6$	Biomatik	See Supplementary Table 3
FtsZ C-terminal peptide	Biomatik	See Supplementary Table 3
FtsZ C-terminal peptide - TAMRA	Biomatik	See Supplementary Table 3
CLPETGG	Biomatik	See Supplementary Table 3
Other		
Pierce™ BCA Protein Assay Kit	ThermoFisher Scientific	23227
Pierce™ Coomassie (Bradford) Protein Assay Kit	ThermoFisher Scientific	23200
PD10 desalting column	Sigma Aldrich	GE17-0851-01l
QCM-D sensor	Biolin Scientific	QSX303
Precision cover glasses thickness No. 1.5H, 24 x 50 mm	VWR (Marienfeld)	107222
Norland Optical Adhesive 63, 1OZ.	APM Technica	284239
HiLoad 26/600 Superdex 200 Prep Grade	GE Healthcare	28-9893-36
Superdex 200 Increase 10/300 GL	GE Healthcare	28-9909-44
Monolith NT.115 Premium Capillaries	Nanotemper	MO-K025
Hellma® fluorescence cuvettes, ultra Micro	Sigma Aldrich	Z802549-1EA
Spectrophotometer Spectramax M2e Plate- + Cuvette Reader	Molecular Devices	
Dyes		
Sulfo-Cyanine-5-maleimide	Lumiprobe	23380
Sulfo-Cyanine-3-maleimide	Lumiprobe	21380
Alexa Fluor® 488 C5 Maleimide	ThermoFisher Scientific	A10254
Software and algorithms		
Fiji/ImageJ		https://fiji.sc
Python (Jupyter Notebook/Spyder)		https://www.anaconda.com/
Astra Software (SEC-MALS)		https://www.wyatt.com
MO. Affinity Analysis Software		https://nanotempertech.com

Supplementary Table 3_Peptide Sequences & Protein Parameters

**Peptide**	**Sequence**	**MW [Da]**
FtsNcyto-His6	CMAQRDYVRRSQPAPSRRKKSTSRKKQRNLPAVHHHHHH	4723.2
FtsZ C-terminal peptide	Ac-KEPDYLDIPAFLRKQAD-NH2	2060.5
FtsZ C-terminal peptide TAMRA	5-TAMRA-KEPDYLDIPAFLRKQAD-NH2	2430.75
CLEPTGG for sortagging	CLEPTGG	675.75

**Protein**	**MW [Da]**	**Width**	**Length**	**pI**
FtsZ	45329.97	~5nm	~7nm	5.84
FtsA WT	45359.99	~5nm	~7nm	5.75
FtsA R286W	40192.72	~4nm	~6nm	4.63
