# Supplementary material for: In vitro reconstitution of Escherichia coli divisome activation
Source: Nat Commun. 2022 May 12;13:2635. doi: 10.1038/s41467-022-30301-y (PMC9098913; doi:10.1038/s41467-022-30301-y)
Supplement: Supplementary file 3 — Description of additional Supplementary File [file 41467_2022_30301_MOESM3_ESM.pdf]

Descriptions of Additional Supplementary Data Files

Supplementary Movie 1

Colocalization of Alexa488-FtsZ (grey; A488-FtsZ) with Cy5-FtsA WT (green). Below 0.4µM, FtsA WT colocalizes well with treadmilling FtsZ filaments. At higher concentrations, FtsA WT remains homogeneously distributed on the membrane. The concentration of FtsZ was kept at 1.25µM. Time lapse movies were acquired at 2s per frame and correspond to Fig. 1c and 1e.

Supplementary Movie 2

Comigration of Cy5-FtsA WT (green) with treadmilling Alexa488-FtsZ filaments (grey). Below 0.4µM, FtsA WT co-migrates well with FtsZ filaments, but fails to do so at higher concentrations. The concentration of FtsZ was kept at 1.25µM. Time lapse movies were acquired at 2s per frame and correspond to Fig. 1d and 1f.

Supplementary Movie 3

Colocalization of lexaA488-FtsZ (grey) with Cy5-FtsA R286W (cyan). FtsA R286W colocalizes well with treadmilling FtsZ filaments at all tested concentrations. The concentration of FtsZ was kept at 1.25µM. Time lapse movies were acquired at 2s per frame and correspond to Fig. 1e and 1g.

Supplementary Movie 4

Comigration of Cy5-FtsA R286W (cyan) with treadmilling Alexa488-FtsZ filaments (grey). FtsA R286W co-migrates well with FtsZ filaments at all tested concentrations. The concentration of FtsZ was kept at 1.25µM. Time lapse movies were acquired at 2s per frame and correspond to Fig. 1f and 1h.

Supplementary Movie 5

Recruitment of Cy5-FtsNcytoHis towards Alexa488-FtsZ filaments at increasing concentrations of FtsA WT. After the addition of 0.1 and 0.2µM FtsA WT with FtsZ, FtsNcytoHis closely follows treadmilling FtsZ filaments. At higher FtsA wt concentrations, the colocalization is lost and FtsNcytoHis covers the membrane homogeneously. The experiments were performed with 0.25% Tris-NTA lipids and 1.25 µM FtsZ. Time lapse movies were acquired at 2s per frame and correspond to Fig. 2f and Fig. S2c.

Supplementary Movie 6

Recruitment of Cy5-FtsNcytoHis towards Alexa488-FtsZ filaments at increasing concentrations of FtsA R286W. After the addition of FtsA R286W and FtsZ, FtsNcytoHis closely follows treadmilling FtsZ filament. The experiments were performed with 0.25% Tris-NTA lipids and 1.25 µM FtsZ. Time lapse movies were acquired at 2s per frame and correspond to Fig. 2f and Fig. S2d.

Supplementary Movie 7

Recruitment of Cy5-FtsNcytoHis towards FtsZ filaments using increasing concentrations of Cy3-FtsA WT. Below 0.2µM FtsA WT, FtsNcytoHis is recruited to FtsZ-FtsA cofilaments. At higher concentrations, FtsA and FtsNcytoHis are distributed homogenously on the membrane. The experiments were performed with 0.25% Tris-NTA lipids and 1.25 µM FtsZ. Time lapse movies were acquired at 2s per frame and correspond to Fig. S2a.

Supplementary Movie 8

Recruitment of Cy5-FtsNcytoHis towards FtsZ filaments using increasing concentrations of Cy3-FtsA R286W. FtsA R286W and FtsNcyto form co-filaments at all concentrations tested. The experiments were performed with 0.25% Tris-NTA lipids and 1.25 µM FtsZ. Time lapse movies were acquired at 2s per frame and correspond to Fig. S2b.

Supplementary Movie 9

Representative single molecule trajectories of Cy5-FtsNcytoHis6 in the presence of 0.2µM FtsA WT or FtsA R286W and 1.25µM FtsZ. The peptide displays diffusive behavior which is interrupted by transient confinement events, where FtsNcytoHis6 binds to FtsA/FtsZ co-filaments. For visualization purposes only one trajectory, which lasts the whole duration of the movie, is shown. The experiments were performed with 1µM unlabeled and 50pM Cy5-FtsNcytoHis6 on membranes containing 0.25% Tris-NTA lipids. The acquisition rate is 51ms and the trajectories correspond to Fig. S2k.

Supplementary Movie 10

Comparison of single molecule dynamics of FtsA WT and R286W at increasing concentrations. While FtsA WT is immobile at concentrations above 0.2µM, FtsA R286W stays diffusive. Additionally, FtsA WT stays membrane bound longer compared to FtsA R286W. The concentration of Cy5-FtsAs is kept constant at 50pM, while the bulk concentration of unlabeled FtsA is increased. Time lapse movies were acquired at 125ms and correspond to Fig. 3a and 3b.

Supplementary Movie 11

Representative movies for FRET experiments of Cy3- and Cy5-labelled FtsA WT in the presence of 1.25µM FtsZ. Upon bleaching Cy5-FtsA WT, a corresponding intensity increase in Cy3-FtsA is observed. FtsA WT only forms filaments below 0.4µM, whereas it remains homogeneously distributed at higher concentrations. Fluorescence recovery is slow and the FRET is strong and long-lasting. Time lapse movies were acquired at 1 frame per second and correspond to Fig. 3h.

Supplementary Movie 12

Representative movies for FRET experiments of Cy3- and Cy5-labelled FtsA R286W in the presence of 1.25µM FtsZ. Upon bleaching Cy5-FtsA R286W, a corresponding intensity increase in Cy3-FtsA R286W is observed. FtsA R286W forms filaments at all concentrations measured. Fluorescence recovery is fast and FRET is short-lived. Time-lapse movies were acquired at 1 frame per second and correspond to Fig. 3h.

Supplementary Movie 13
Representative movies for FRET experiments of 0.4µM FtsA R286W alone, with FtsNcytoHis and with FtsNcytoHis and 1.25µM FtsZ. The measured FRET increases significantly in the presence of interaction partners. Fluorescence recovery is also slowed down, but it is still significantly faster than for FtsA WT. The movies are representative of one experiment, where the components where added sequentially. The experiment was performed at 0.25% Tris-NTA membranes and acquired at 2 frames per second and correspond to Fig. 3h.

Supplementary Movie 14
Colocalization of Cy5-FtsA-His6 with treadmilling Alexa488-FtsZ filaments at increasing Tris-NTA lipid ratios. FtsA-His6 colocalizes well with FtsZ filaments at densities up to 1% Tris-NTA lipids. At higher densities, FtsA-His6 fails to colocalize with FtsZ filaments and forms a homogeneous layer on the membrane. The concentration of FtsZ was kept at 1.25µM. Time lapse movies were acquired at 2s per frame and correspond to Fig. 4a and 4c.

Supplementary Movie 15
Colocalization of Cy5-FtsA R286W-His6 with treadmilling Alexa488-FtsZ filaments at increasing Tris-NTA lipid ratios. To form a stable pattern, the density of Tris-NTA lipids needs to be higher than 0.6%. FtsA R286W-His6 colocalizes well with FtsZ filaments at densities up to 2.0% Tris-NTA lipids. The concentration of FtsZ was kept at 1.25µM. Time lapse movies were acquired at 2s per frame and correspond to Fig. 4b and 4c.

Supplementary Movie 16
Comparison of FRET experiments of His-tagged versions of FtsA WT and FtsA R286W at membranes with 1.0% and 2.0% Tris-NTA in the presence of 1.25µM unlabelled FtsZ. The recovery of both proteins is slowed down compared to the native versions of the protein and is dominated by lateral diffusion. FtsA R286W-His6 still recovers faster than FtsA WT and the measured FRET increase is weaker, indicated by the lower intensity increase in the FRET channel. Time lapse movies were acquired at 2s per frame and correspond to Fig. 4e, 4f and 4g.

Supplementary Movie 17

Colocalization of Cy5-FtsA-His6 and R286W-His6 with Alexa488-FtsZ in the presence of $FtsN_{cyto}$ at a Tris-NTA lipid density of 1.5%. While both His tagged FtsAs are able to colocalize with FtsZ, the dynamics of the pattern are dramatically decreased. Time lapse movies were acquired at 2s per frame and correspond to Fig. S5a and S5b.

Supplementary Movie 18

Comparison of FRET experiments of His-tagged versions of FtsA WT and FtsA R286W at membranes with 1.5% in the presence of 1.25µM unlabelled FtsZ and $FtsN_{cyto}$. The recovery of both proteins dramatically decreased and both variants do not recover within the time of the experiment. The measured FRET is similar for both His tagged FtsAs in the presence of $FtsN_{cyto}$. Time lapse movies were acquired at 2s per frame and correspond to Fig. S5c-S5h.
