# Supplementary material for: In vitro reconstitution of Escherichia coli divisome activation
Source: Nat Commun. 2022 May 12;13:2635. doi: 10.1038/s41467-022-30301-y (PMC9098913; doi:10.1038/s41467-022-30301-y)
Supplement: Supplementary file 22 — Reporting Summary [file 41467_2022_30301_MOESM22_ESM.pdf]

nature portfolio

Corresponding author(s): Martin Loose

Last updated by author(s): Apr 4, 2022

Reporting Summary

Nature Portfolio wishes to improve the reproducibility of the work that we publish. This form provides structure for consistency and transparency in reporting. For further information on Nature Portfolio policies, see our Editorial Policies and the Editorial Policy Checklist.

Statistics

For all statistical analyses, confirm that the following items are present in the figure legend, table legend, main text, or Methods section.

n/a	Confirmed	
☐	☒	The exact sample size (*n*) for each experimental group/condition, given as a discrete number and unit of measurement
☐	☒	A statement on whether measurements were taken from distinct samples or whether the same sample was measured repeatedly
☐	☒	The statistical test(s) used AND whether they are one- or two-sided<br>*Only common tests should be described solely by name; describe more complex techniques in the Methods section.*
☒	☐	A description of all covariates tested
☒	☐	A description of any assumptions or corrections, such as tests of normality and adjustment for multiple comparisons
☐	☒	A full description of the statistical parameters including central tendency (e.g. means) or other basic estimates (e.g. regression coefficient) AND variation (e.g. standard deviation) or associated estimates of uncertainty (e.g. confidence intervals)
☒	☐	For null hypothesis testing, the test statistic (e.g. *F*, *t*, *r*) with confidence intervals, effect sizes, degrees of freedom and *P* value noted<br>*Give P values as exact values whenever suitable.*
☒	☐	For Bayesian analysis, information on the choice of priors and Markov chain Monte Carlo settings
☒	☐	For hierarchical and complex designs, identification of the appropriate level for tests and full reporting of outcomes
☒	☐	Estimates of effect sizes (e.g. Cohen's *d*, Pearson's *r*), indicating how they were calculated

Our web collection on statistics for biologists contains articles on many of the points above.

Software and code

Policy information about availability of computer code

Data collection	No software was used for data collection.
Data analysis	For the analysis of the microscopy data ImageJ Version v1.53p and Jupyter Notebook (6.4.8) were used. MST data was analyzed with the MO.Affinity Analysis software and SEC-MALS data was analyzed with Wyatts ASTRA software.<br>For analysis of FtsN confinement and the FRAP analysis custom built codes were implemented. The codes are found in https://doi.org/10.5281/zenodo.6397261 and https://doi.org/10.5281/zenodo.6400639 respectively. For analysis of FtsZ treadmilling, we used a previously published code, which can be found at https://doi.org/10.5281/zenodo.6397778.

For manuscripts utilizing custom algorithms or software that are central to the research but not yet described in published literature, software must be made available to editors and reviewers. We strongly encourage code deposition in a community repository (e.g. GitHub). See the Nature Portfolio guidelines for submitting code & software for further information.

Data

Policy information about availability of data

All manuscripts must include a data availability statement. This statement should provide the following information, where applicable:

- Accession codes, unique identifiers, or web links for publicly available datasets
- A description of any restrictions on data availability
- For clinical datasets or third party data, please ensure that the statement adheres to our policy

Source data for Figs 1-4 and Supplementary Figures 1-5 are provided with the paper. Additionally we uploaded all raw microscopy images together with Jupyter Notebooks used to plot the data to https://research-explorer.app.ist.ac.at/librecat/record/preview/10934.

Field-specific reporting

Please select the one below that is the best fit for your research. If you are not sure, read the appropriate sections before making your selection.

- [x] Life sciences
- [ ] Behavioural & social sciences
- [ ] Ecological, evolutionary & environmental sciences

For a reference copy of the document with all sections, see nature.com/documents/nr-reporting-summary-flat.pdf

Life sciences study design

All studies must disclose on these points even when the disclosure is negative.

Sample size	Sample sizes were not chosen according to a statistical test, but were limited by practicality and throughput.
Data exclusions	Images were only excluded when supported lipid bilayers were defective.
Replication	The results were verified and reproduced with different batches of purified FtsZ and FtsA proteins. All experiments were performed at least in two or more independent experiments with at least one sample chamber each under the same conditions, so that the total sample number was always ≥2.
Randomization	This study does not include work with participants or living organisms.
Blinding	This study does not include work with participants or living organisms.

Reporting for specific materials, systems and methods

We require information from authors about some types of materials, experimental systems and methods used in many studies. Here, indicate whether each material, system or method listed is relevant to your study. If you are not sure if a list item applies to your research, read the appropriate section before selecting a response.

Materials & experimental systems

n/a	Involved in the study	
x		Antibodies
x		Eukaryotic cell lines
x		Palaeontology and archaeology
x		Animals and other organisms
x		Human research participants
x		Clinical data
x		Dual use research of concern

Methods

n/a	Involved in the study	
x		ChIP-seq
x		Flow cytometry
x		MRI-based neuroimaging
